# Dynamic interplay of multidrug transporters with TolC for isoprenol tolerance in *Escherichia coli*

**DOI:** 10.1038/srep16505

**Published:** 2015-11-13

**Authors:** Chonglong Wang, Liyang Yang, Asad Ali Shah, Eui-Sung Choi, Seon-Won Kim

**Affiliations:** 1 Division of Applied Life Science (BK21 Plus), PMBBRC, Gyeongsang National University, Jinju 660-701, Republic of Korea; 2 Industrial Biotechnology Research Center, KRIBB, Daejeon 305-806, Republic of Korea

## Abstract

Engineering of efflux pumps is a promising way to improve host’s tolerance to biofuels such as medium-chain alcohols (C_m_OHs); however, this strategy is restricted by poor understanding of the efflux pumps engaged in extrusion of solvents. In this study, several *Escherichia coli* mutants of multidrug transporters were evaluated for isoprenol tolerance. Susceptible phenotypes were observed in the mutants with individual deletion of six transporters, AcrD, EmrAB, MacAB, MdtBC, MdtJI and YdiM, whereas inactivation of AcrAB transporter resulted in an improved tolerance to isoprenol and other C_m_OHs. AcrAB is the major transporter forming tripartite transperiplasmic complex with outer membrane channel TolC for direct extrusion of toxic molecules in *E*. *coli*. The AcrAB inactivation enables to enhance TolC availability for the multidrug transporters associated with extrusion of C_m_OHs and increase the tolerance to C_m_OHs including isoprenol. It is assumed that outer membrane channel TolC plays an important role in extrusion of isoprenol and other C_m_OHs.

The development of microbial systems for biofuel production has gained significant interest owing to the rising concerns about sustainability of fossil fuels and global climate changes. With the advent of metabolic engineering and synthetic biology, microbes have been engineered to synthesize various biofuel candidates^1^,^2^,^3^. Medium-chain (C_4_-C_9_) alcohols (C_m_OHs) have a high energy density, and are compatible with the existing infrastructure as compared with ethanol (Supplementary Table S1). These fuel-like alcohols can be blended with gasoline (C_4_-C_12_) and even be used as a drop-in fuel by themselves. A principal challenge in processing bulk production of C_m_OHs is their toxicity to the host^4^. The cytotoxicity may be associated with denaturation of proteins, damage to DNA and lipid, and RNA degradation^5^. To respond to such cytotoxic stresses, microorganisms have evolved a variety of defense strategies including changes in membrane composition, activation of stress response genes and overexpression of efflux pumps^6^,^7^, which inspires biologists to develop a robust workhorse tolerant to the toxic C_m_OHs. As biofuels are produced within cells, efflux pumps would perhaps be the most promising strategy to alleviate their toxicity to cells by pumping them out of microbes (Fig. 1A).

Efflux transporters are a class of membrane proteins that recognize and expel toxic compounds to the extracellular milieu^8^,^9^. Although solvent-specific exporters have been characterized from solvent-resistant species^10^, many transporters can pump out a broad range of substrates including organic solvents^11^. Transporter engineering has been well implemented to improve tolerance of biofuel-producing hosts^12^. However, such an approach is generally restricted to the knowledge whether efflux transporters can recognize and transport target molecules. Moreover, expression of some heterologous transporters suffers from codon bias of microbes and notorious difficulty of membrane protein expression^13^,^14^. These limitations compromise the success in engineering tolerant hosts to biofuels, especially C_m_OHs^12^.

Most microbes contain a large number of intrinsic multidrug transporters (MDTs). Some MDTs exhibit up-regulation behaviors in the response network upon butanol exposure (Supplementary Fig. S1)^15^. It could be possible to identify and modulate native pumps of microbes for desired tolerance phenotypes to C_m_OHs. There are at least 37 putative MDTs found in *Escherichia coli*, belonging to five different families: major facilitator (MF) family, small multidrug resistance (SMR) family, resistance nodulation cell division (RND) family, ATP-binding cassette (ABC) family and multidrug and toxic compound extrusion (MATE) family^16^. Of these, nine transporters have been reported to interact with TolC, an outer membrane (OM) channel protein, to form tripartite transperiplasmic complexes for direct extrusion of toxic compounds out of cells^17^. Production of isoprenol has been achieved in the tractable host *E*. *coli* at a titer of 1.3 g/L (0.15%, v/v) to date^18^. With the continuous effort of metabolic engineering, the biofuel production will be finally limited by hosts’ innate tolerance themselves. To probe native isoprenol transporters, it was assumed that any deletion of MDT gene associated with isoprenol extrusion would result in a reduced tolerance phenotype in comparison with the wild type *E. coli* (Fig. 1A,B). In this scenario, a library of 45 null mutants (Supplementary Table S2) associated with 37 MDTs was thus screened. Six MDTs, AcrD, EmrAB, MacAB, MdtBC, MdtJI and YdiM, were found to be involved in alleviating isoprenol toxicity. Interestingly, the inactivation of AcrAB, the major MDT transporter, significantly improved *E. coli* tolerance to isoprenol. Thus, a possible tolerance mechanism was elucidated in the context of a complicated network of membrane transporters.

## Results and Discussion

### Several multidrug transporter (MDT) mutants are susceptible to isoprenol

**Figure 1:**
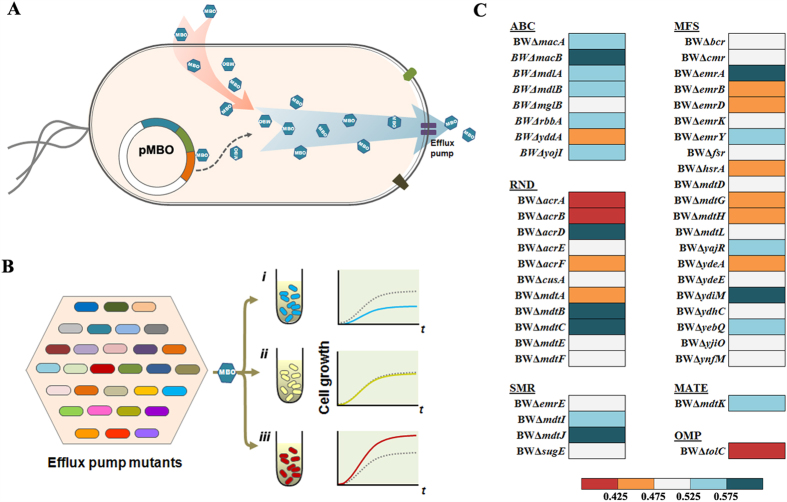
Screening transporters capable of isoprenol extrusion. (**A**) Isoprenol extrusion by transporters. If an efflux pump in membrane is engaged in isoprenol extrusion, it would reduce the toxicity of isoprenol either synthesized endogenously or added exogenously. Blue hexagons labeled with “MBO” represent isoprenol (3M-3 = C_4_OH) molecules. pMBO indicates the plasmid for endogenous isoprenol synthesis. (**B**) Screening strategy of multidrug transporters (MDTs) for isoprenol tolerance. MDT mutants are grown in presence of isoprenol stress. Three cell growth genotypes can be expected: (*i*) if a transporter is involved in isoprenol extrusion, its deletion would exhibit growth inhibition in comparison with the wild type; (*ii*) if not involved, there should be no growth difference between the transporter mutant and the wild type strain; and (*iii*) perhaps, a tolerated phenotype would result from some unknown mechanisms. (**C**) Growth inhibition of mutants by isoprenol. MDT mutants were grown in 2YT medium with 0.5% (v/v) of isoprenol at 30 °C for 12 h. By comparison of growth inhibition (%) of each mutant relative to wild type strain, the mutants were categorized into 5 groups: very susceptible (>57.5%, dark cyan), susceptible (52.5–57.5%, cyan), no different (47.5–52.5%, white), resistant (42.5–47.5%, orange), and very resistant (<42.5%, red). The growth inhibition of the wild type strain is at approximate 50% as a reference. Results are the means of two biological replicates.

**Figure 2:**
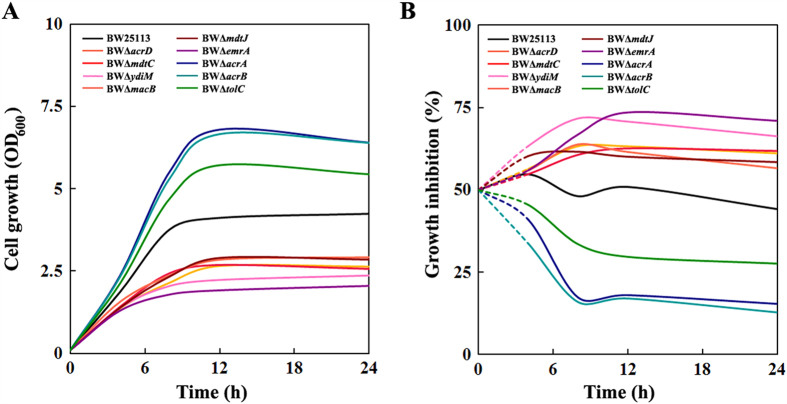
Growth phenotype of isoprenol susceptible and resistant mutants. (**A**) Cell growth of transporter mutants. (**B**) Growth inhibition of transporter mutants. Susceptible mutants BWΔ*acrD* (orange), BWΔ*emrA* (purple), BWΔ*macB* (tomato) BWΔ*mdtC* (red), BWΔ*mdtJ* (maroon) and BWΔ*ydiM* (pink); resistant mutants BWΔ*acrA* (blue), BWΔ*acrB* (cyan) and BWΔ*tolC* (green); and wild type *E*. *coli* BW25113 (black) were grown in 2YT medium with 0.5% (v/v) of isoprenol at 30 °C. Cell growth was measured every 6 h. Growth inhibition was calculated as described in Materials and Methods. Initial growth inhibition was assumed as 50% in all strains. Results are the means of two biological replicates.

**Figure 3:**
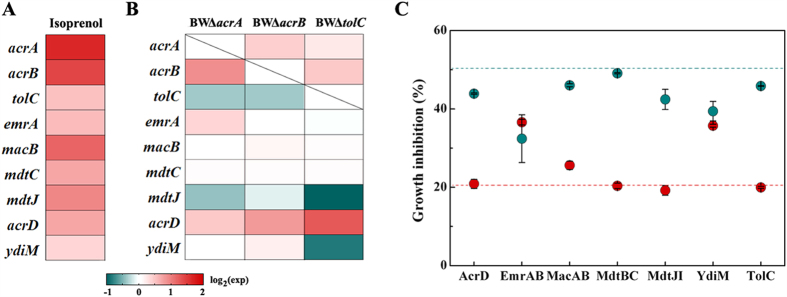
Validation of transporters conferring isoprenol tolerance by quantitative PCR analysis and their overexpression. (**A**) Transcript profile of *E*. *coli* BW25113 upon exposure to isoprenol. *E*. *coli* BW25113 was grown in 2YT medium with 0.5% (v/v) of isoprenol at 30 °C for 6 h. Transcript changes (folds) were normalized to those of the cultures without isoprenol. Results are the means of three biological replicates. (**B**) Transcript profiles of the mutants BWΔ*acrA*, BWΔ*acrB* and BWΔ*tolC*. Three mutants were grown in 2YT medium with 0.5% (v/v) of isoprenol at 30 °C for 6 h. Transcript changes (folds) were normalized to *E*. *coli* BW25113. Results are the means of three biological replicates. The slashed cells indicate that quantitative PCR was not conducted. (**C**) Overexpression of transporters engaged in isoprenol extrusion. Growth inhibition was determined with *E*. *coli* BW25113 (dark cyan dots) and BWΔ*acrAB* (red dots) expressing AcrD, EmrAB, MacAB, MdtABC, MdtJI and YdiM. Growth inhibition of *E*. *coli* BW25113 (dark cyan dashed-line) and BWΔ*acrAB* (red dashed-line) harboring an empty vector pTrc99A with no overexpression of these transporters was also measured as a control. All strains were grown in 2YT medium with 0.5% (v/v) of isoprenol and 0.1 mM of IPTG at 30 °C for 12 h. Error bars represent the standard deviations of two biological replicates.

Isoprenol is an unsaturated C_5_ alcohol with octanol/water partition coefficient (logPo/w) of 1.14. Solvents with logPo/w of 1–5 are generally regarded to be toxic to cells. The cell growth of wild type *E*. *coli* BW 25113 was half inhibited by 0.5% (v/v) of isoprenol. To explore the possible transporters for isoprenol extrusion, cell growth was evaluated after 12 h exposure to 0.5% (v/v) of isoprenol in a total of 44 MDT null mutants (Fig. 1C and Supplementary Table S3). If any mutant exhibits a decreased growth phenotype in comparison with the wild type, it may suggest that this transporter is involved in isoprenol extrusion. As a result, growth inhibition over 52.5% was observed in 17 mutants, that is, they are 5% more susceptible to isoprenol than wild type *E*. *coli* BW 25113. In particular, seven null mutants of 6 MDTs (AcrD, EmrAB, MacAB, MdtBC, MdtJI and YdiM) (Fig. 1C) showed growth inhibition of more than 57.5%, equivalent to 15% higher susceptibility than wild type strain. The time courses of cell growth confirmed that the genetic deficiency in these 6 MDTs indeed resulted in severe growth inhibition when isoprenol was present (Fig. 2), whereas there was no significant difference in growth in the absence of isoprenol (Supplementary Fig. S2). The genetic response of the wild type *E*. *coli* BW 25113 upon isoprenol stress was analyzed by quantitative PCR (Fig. 3A and Supplementary Table S4). Isoprenol exposure induced up-regulation of all the six MDTs genes, of which *macB* showed a 2.4-fold increase in the transcription level. Taken together, these results validate that these 6 MDTs may act on isoprenol extrusion and alleviate the toxicity to *E. coli*. In accordance with the survival mechanism of *E*. *coli*^19^, several MDTs appeared to be involved in extrusion of isoprenol, which provides *E*. *coli* with an effective strategy to challenge toxic isoprenol environment. These identified MDTs were consequently overexpressed in *E. coli* BW25113 to see whether their expression improves the resistance to isoprenol (Fig. 3C and Supplementary Fig. S3). Expression of these MDTs except MdtBC improved the isoprenol tolerance in *E*. *coli*, *i*.*e*. EmrAB-expressing strain just exhibits 32.4% of growth inhibition to 0.5% (v/v) of isoprenol, representing a 1.4-fold increase to wild type BW25113. It is generally accepted that membrane proteins are expressed at a low level due to the spatially-delimited membranous environment, which may compromise the tolerance improvement in these strains. Indeed, the plasmid-based overexpression of transporters did not lead to legible protein band in SDS-PAGE gel among strains (Supplementary Fig. S4).

### Inactivation of AcrAB transporter confers an improved isoprenol tolerance upon *E*. *coli*

**Figure 4:**
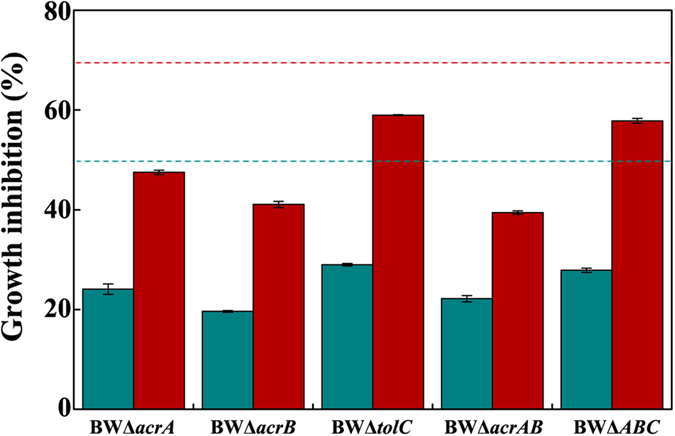
Role of TolC in isoprenol extrusion. All mutants were grown in 2YT medium with 0.5% (v/v) (dark cyan bars) or 0.75% (v/v) (red bars) of isoprenol at 30 °C for 12 h. Dashed-lines with dark cyan and red colors represent growth inhibition of wild type *E*. *coli* BW25113 by 0.5% (v/v) and 0.75% (v/v) of isoprenol, respectively. Error bars represent the standard deviations of two biological replicates.

Interestingly, eleven MDT mutants exhibited phenotypes with higher tolerance to isoprenol than wild type *E*. *coli* BW 25113 (Fig. 1C and Supplementary Fig. S3). Of the two most resistant mutants BWΔ*acrA* and BWΔ*acrB*, the addition of 0.5% (v/v) of isoprenol just resulted in less growth inhibition of around 23% (Figs 1C and 2), indicating a 1.6-fold increase in tolerance capacity when compared with wild type BW25113. In accordance with our observation, mutations at *acrAB* locus conferring an improved tolerance have been also identified from isobutanol-adaptive *E*. *coli* strains^20^,^21^. Thus, mRNA levels of transporters were subsequently quantified to see whether the deletion of *acrA* or *acrB* increases the transcription of the six MDTs identified to increase the tolerance (Fig. 3B and Supplementary Table S4). However, there was just no more than 0.8-fold increase in transcription of *acrD* gene and no significant changes in the other MDT genes between the mutants (BWΔ*acrA* and BWΔ*acrB*) and the wild type *E*. *coli* BW 25113. The double knockout mutant BWΔ*acrAB* was also created to investigate whether the double deletion of *acrA* and *acrB* could show an additive tolerance, but the mutant BWΔ*acrAB* did not outperform the individual mutants BWΔ*acrA* and BWΔ*acrB* (Fig. 4). Thus, the enhanced tolerance to isoprenol in the mutants BWΔ*acrA* and BWΔ*acrB* is most likely due to the inactivation of AcrAB transporter, not an increase in expression of the six MDTs transporters.

### Isoprenol may be transported through both TolC-dependent and TolC-independent routes

The RND transporter AcrAB is generally assembled with the outer membrane (OM) channel protein TolC to form a tripartite AcrAB-TolC complex expelling drugs from the cell interior^22^. Universal TolC is also shared by various other transporters in *E*. *coli* including AcrD, MacAB, EmrAB and MdtBC, whose deletion herein results in *E*. *coli* susceptible to isoprenol. It suggests that TolC might interplay with the transporters involved in isoprenol tolerance. Thus, tolerance phenotype of mutant BWΔ*tolC* was studied in presence of 0.5% (v/v) of isoprenol (Figs 1C and 2). The deletion of *tolC* unexpectedly showed an improved isoprenol tolerance, albeit not as effective as the deletion of *acrA* or *acrB*. The growth inhibition of 33.9% was observed in *E*. *coli* BWΔ*tolC*, presenting 133.5% of the relative tolerance to *E*. *coli* BW25113. This result reveals that isoprenol is expelled in a TolC-independent route from the cell interior. It is assumed that isoprenol is exported into the periplasm by some transporters which are able to cooperate with other OM channels beside TolC for isoprenol extrusion from periplasm to the cell exterior. The deletion of *tolC* is reported to be accompanied with an increased expression of other OM porins^23^, which may be related to the tolerance improvement in *E*. *coli* BWΔ*tolC*. Moreover, mRNA level of some MDTs such as *acrD* was increased by 2.4-fold in the mutant BWΔ*tolC* as compared with wild type BW25113 (Fig. 3B). Such an increase in MDT transcription indicates that some potential transporters may serve for improvement of isoprenol tolerance in the mutant BWΔ*tolC*.

Moreover, it should be noted that the mutant BWΔ*tolC* was less tolerant to isoprenol than the mutants BWΔ*acrA*, BWΔ*acrB* and BWΔ*acrAB* (Fig. 2 and 4). *E*. *coli* BWΔ*tolC* exhibits 88.3% of the tolerance capacity of *E*. *coli* BWΔ*acrB*. The tolerance difference between these two strains was more apparent when isoprenol concentration was increased to 0.75% (v/v). The tolerance capacity of the mutant BWΔ*tolC* was 69.6% of that of the mutant BWΔ*acrB* (Fig. 4). To explore whether a combined deletion of AcrAB and TolC can further increase isoprenol tolerance, the triple knock-out mutant BWΔ*ABC* was constructed and cultivated in presence of isoprenol. If deletions of AcrAB and TolC confer isoprenol tolerance by independent mechanisms, the mutant BWΔ*ABC* would exhibit an additional tolerance. However, the mutant BWΔ*ABC* tolerance was similar to that of the mutant BWΔ*tolC* (Fig. 4), and the complementary expression of TolC by plamid pT-tolC could restore the tolerance comparable to that of the mutant BWΔ*acrAB* (Supplementary Fig. S5). This result suggests that the dedicated tolerance by the AcrAB knock-out is dependent on TolC.

TolC exhibits affinities to various transporters and is able to interact with them in a very dynamic process^24^. The transporters would compete with each other for recruitment of TolC, which can be affected by their abundance. AcrAB transporter is a major MDT and constitutively expressed at a higher level than other transporters in *E. coli*^25^,^26^. Quantitative PCR analysis showed that *acrB* was transcribed at a much higher level (>6.5 folds) than the identified six MDTs transporters regardless of whether exposed to isoprenol or not (Supplementary Fig. S6). The transcription of *acrAB* operon was even enhanced by isoprenol exposure (Fig. 3A). Thus, it is assumed that the dominant AcrAB transporter molecules occupy most of TolC molecules in wild type *E. coli* BW25113, whereas TolC molecules become more available for non-dominant transporters such as AcrD, MacAB, EmrAB and MdtBC in the AcrAB knock-out mutant. In this assumption, the transporters engaged in isoprenol extrusion are able to recruit TolC in an easier way in the mutants BWΔ*acrA*, BWΔ*acrB* and BWΔ*acrAB*, which improves the tolerance to isoprenol.

However, the tolerance is not further increased by TolC overexpression in the wild type *E*. *coli* BW25113 and the mutant BWΔ*acrAB* (Fig. 3C) because TolC is intrinsically expressed at a significant level in a spatially-delimited OM (Supplementary Fig. S6)^26^. Overexpression of the identified six MDTs does not give a further improvement of isoprenol tolerance in the mutant BWΔ*acrAB* as well (Fig. 3C). It is also likely due to the spatially-delimited membranous environment which restricts overexpression of the transporters. However, it remains to be elucidated the exquisite interplay mechanism between TolC and the associated transporters for comprehensive understanding of isoprenol tolerance among the MDTs knockout mutants, which would be a principal guide in transporter engineering for biofuel tolerance. Taken together, the deletion *acrAB* encoding a “useless” transporter for isopentenol extrusion allows more membranous space and available TolC for the associated transporters with isoprenol extrusion.

### Inactivation of AcrAB transporter also exhibits general tolerance to other medium-chain alcohols (C_m_OHs)

**Figure 5:**
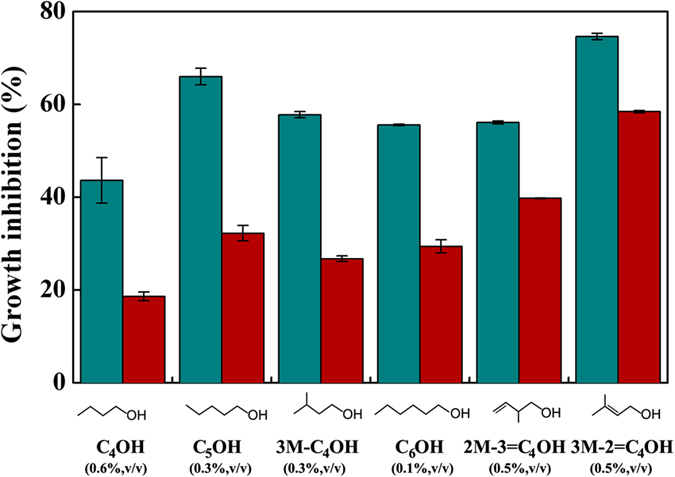
Deletion of AcrAB enhances tolerance to various medium-chain alcohols. *E. coli* BW25113 (dark cyan bars) and BWΔ*acrAB* (red bars) were grown in 2YT medium with various C_m_OHs at a given concentration (%, v/v) at 30 °C for 12 h. Error bars represent the standard deviations of two biological replicates.

*E*. *coli* BWΔ*acrAB* was grown in presence of various C_m_OHs with different chemical structures and properties (Supplementary Table S1) to investigate whether the inactivation of AcrAB transporter provides tolerance to other C_m_OHs. *E*. *coli* BWΔ*acrAB* possesses higher tolerance capacity to all tested C_m_OHs than wild type BW25113 (Fig. 5). A microarray analysis was used to study the response of transporters to butanol^15^. It reported up-regulation of several transporters upon exposure to butanol (Supplementary Fig. S1), but the up-regulated transporters are not identical to the transporters observed under isoprenol exposure (Fig. 3A). Thus, it is speculated that *E. coli* uses different transporters for extrusion of different C_m_OHs because transporters generally have their own substrate preferences. However, the transporters have to cooperate with the universal TolC to form transperiplasmic channels to effectively expel C_m_OHs. The availability of TolC for transporters becomes so crucial to enhance tolerance to C_m_OHs. The inactivation of AcrAB can liberate TolC for various transporters induced by specific C_m_OHs, and finally endow *E*. *coli* with desirable biofuel tolerance.

## Conclusions

**Figure 6:**
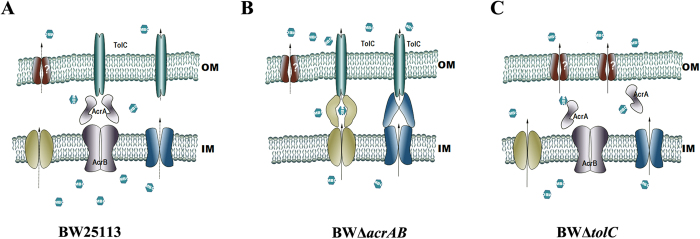
Proposed mechanisms for isoprenol extrusion in *E. coli*. (**A**) Isoprenol extrusion in wild type *E. coli* BW25113. The main AcrAB transporter is located in inner membrane (IM), and generally forms a tripartite complex with outer membrane (OM) channel TolC. However, the complex does not act on isoprenol extrusion. Some other transporters such as AcrD, EmrAB, MacAB, MdtABC, MdtJI and YdiM are able to expel isoprenol, but their capability expelling isoprenol is limited by TolC availability and low abundance of the transporters. (**B**) Isoprenol extrusion in the mutant *E. coli* BWΔ*acrAB*. The deletion of *acrAB* encoding AcrAB transporter increases the availability of TolC for isoprenol transporters to form transporter-TolC complexes for direct extrusion of isoprenol. (**C**) Isoprenol extrusion in the mutant *E. coli* BWΔ*tolC*. The transporter cannot form the transperiplasmic complex due to no TolC in the mutant *E. coli* BWΔ*tolC.* Transporters may work individually and expel isoprenol into the periplasm, and some channels (question mark) other than TolC may transport isoprenol out in a TolC-independent manner. This TolC-independent route functions less effectively than the TolC-dependent route for isoprenol extrusion.

A myriad of MDTs provide a survival strategy against various toxic compounds, which inspires metabolic engineers to use the MDTs to relieve biofuel toxicity and to increase the yield in a biological process. In this study, a number of null MDT mutants were surveyed under isoprenol stress. The null mutants of transporters AcrD, EmrAB, MacAB, MdtBC, MdtJI and YdiM were more susceptible (>15%) to isoprenol than wild type *E*. *coli* BW25113 (Figs 1C and 2). This result suggests a possibility to develop an isoprenol resistant host by engineering the transporters. It is also interesting to find that the inactivation of AcrAB leads to a significant increase in tolerance capacity to isoprenol (Figs 1C and 2). The major transporter AcrAB is not involved in isoprenol extrusion, but occupies most TolC, a crucial OM channel protein cooperating with various MDTs for extrusion of toxic chemicals (Fig. 6A). Thus, the deletion of AcrAB liberates TolC to form transperiplasmic channels with the identified 6 MDTs (AcrD, EmrAB, MacAB, MdtBC, MdtJI and YdiM) for effective isoprenol extrusion (Fig. 6B). On the other hand, *E*. *coli* BWΔ*tolC* also exhibited an improved isoprenol tolerance over the wild type *E*. *coli* BW25113 (Figs 1C and 2), suggesting that isoprenol can be exported by a TolC-independent route (Fig. 6C), albeit less effective than the TolC-dependent route. An increase in available TolC for the transporters can be generalized to most of the C_m_OHs expelled by the TolC-dependent route, although the desired transporters may differ among C_m_OHs (e.g. butanol vs. isoprenol). Conclusively, our results provide a new insight into the tolerance engineering of hosts for mass production of the fuel-like alcohols.

## Materials and Methods

### Chemicals and reagents

Restriction enzymes and T4 DNA ligase were purchased from New England Biolabs (Beverly, MA). Custom oligodeoxynucleotides were synthesized by Bioneer (Daejoen, Korea). RNA extraction and quantitative PCR kits were ordered from Qiagen (Seoul, Korea). Isoprenol, 1-butanol, 1-isopentanol, 1-pentanol and 1-hexanol are products of Sigma-Aldrich (St. Louis, MO).

### Strains and culture condition

*E. coli* DH5α (F^−^, λ^−^, *endA*1, *glnV*44, *thi*-1, *recA*1, *relA*1, *gyrA*96, *deoR*, *Φ*80, d*lac*ZΔM15, Δ(l*acZYA*-*argF*) U169, *hsd*R17(r_K_^−^ m_K_^+^), *supE*44) was used as a host for gene cloning. *E*. *coli* BW25113 (F^−^, λ^−^, *rrnB*-3, Δ*lacZ*4787, Δ(*araD*-*araB*)567, *hsd*R514, Δ(*rhaD*-rha*B*) 568, *rph*-1) and its isogenic deletion mutants were obtained from Keio collection (National Institute of Genetics, Shizuoka, Japan) for screening of transporter candidates associated with isoprenol tolerance. *E*. *coli* BWΔ*acrAB* and BWΔ*ABC* mutants were created by λ Red-mediated recombination method using *E*. *coli* BW25113 and *E*. *coli* BWΔ*tolC* as parent strains, respectively. A kanamycin resistance cassette for *acrAB* deletion was amplified from plasmid pKD13 using primers DacrAB-F and DacrAB-R (Supplementary Table S5). Recombination was performed according to the literature^27^. Before the recombination in *E*. *coli* BWΔ*tolC*, the kanamycin resistance cassette of *tolC* allele was cured by FLP recombinase expressed from plasmid pCP20. All mutants used in this work are listed in Table S2.

Cells were grown at 37 °C or 30 °C in 2YT medium (Bacto-tryptone 16 g, Bacto-yeast extract 10 g, and sodium chloride 5 g per liter, adjusted to pH 7.0) with agitation. Kanamycin (50 μg/mL) and ampicillin (100 μg/mL) were added as required. Cell density was measured on Spectrophotometer DU 730 (Beckman Coulter, Seoul, Korea) at 600 nm (OD_600_).

### Determination of growth inhibition, and relative susceptibility and tolerance capacities

Overnight culture was inoculated at an optical density (OD_600_) of 0.1 into a PYREX^®^55 mL rimless culture tube (Corning, NY) containing 4 mL of fresh media with (or without) isoprenol or other C_m_OHs at a given concentration (%, v/v). Cultivations were carried out at 30 °C and cell growth (OD_600_) was determined at given culture time. Growth inhibition (%) is defined as (1 − OD_600 with CmOHs_ / OD_600 without CmOHs_) × 100. Relative susceptibility (Strain A to Strain B, %) is calculated by (growth inhibition of Strain A/growth inhibition of Strain B) × 100, and relative tolerance capacity (%) by [(1− growth inhibition of Strain A)/(1− growth inhibition of Strain B)] × 100.

### Quantitative PCR analysis

Total RNA was isolated from *E*. *coli* BW25113 and mutant strains by using RNeasy® mini Kit (Qiagen, Seoul, Korea) according to the manufacturer’s instruction. The isolated RNA was subjected to additional DNase digestion by using RNase-Free DNase Set (Qiagen, Seoul, Korea). Reverse transcription and Quantitative PCR were performed with Rotor-Gene^TM^ SYBR® Green RT-PCR Kit (Qiagen, Seoul, Korea) in Rotor-gene® Q cycler (Qiagen, Seoul, Korea). The *cysG* was used as the housekeeping gene for quantification of targeted transcript changes. Primers used for quantitative PCR are listed in Supplementary Table S6.

### Construction of plasmids

Genes of *acrD*, *emrAB*, *macAB*, *mdtBC*, *mdtJI* and *ydiM* were amplified from genomic DNA of *E*. *coli* BW25113 by using each primer set (Supplementary Table S7). The resulting PCR products of *acrD*, *emrAB*, *mdtJI* and *ydiM* were digested with BamHI and SalI, and cloned into the BamHI-SalI sites of pTrc99A to create pT-acrD, pT-emrAB, pT-mdtJI and pT-ydiM. The *mdtBC* product was digested with BglII and XhoI, and also cloned into the BamHI-SalI sites of pTrc99A to create pT-mdtBC. The *macAB* product digested with KpnI and XbaI was cloned into the corresponding restriction sites of pTrc99A to create pT-macAB. Plasmids used in this study are listed in Supplementary Table S8.

## Additional Information

**How to cite this article**: Wang, C. *et al.* Dynamic interplay of multidrug transporters with TolC for isoprenol tolerance in *Escherichia coli*. *Sci. Rep.* **5**, 16505; doi: 10.1038/srep16505 (2015).

## Supplementary Material

Supplementary Information
