# Supplementary material for: Dynamic interplay of multidrug transporters with TolC for isoprenol tolerance in Escherichia coli
Source: Sci Rep. 2015 Nov 13;5:16505. doi: 10.1038/srep16505 (PMC4643228; doi:10.1038/srep16505)
Supplement: Supplementary Information [file srep16505-s1.pdf]

Supplementary information for

Dynamic interplay of multidrug transporters with TolC for isoprenol tolerance in *Escherichia coli*

Chonglong Wang[1], LiyangYang[1], Asad Ali Shah[1], Eui-Sung Choi[2*] and Seon-Won Kim[1*]

[1]Division of Applied Life Science (BK21 Plus), PMBBRC, Gyeongsang National University, Jinju 660-701, Republic of Korea

[2]Industrial Biotechnology Research Center, KRIBB, Daejeon 305-806, Republic of Korea

*Correspondence and requests for materials should be addressed to Tel.: +82 55 772 1362; Fax: +82 55 759 9363. E-mail: swkim@gnu.ac.kr (S. -W. Kim) or Tel.: +82 42 860 4453; Fax: +82 42 860 4489; E-mail: choi4162@kribb.re.kr (E. -S. Choi)

Table S1. Chemical and physical properties of medium-chain alcohols, gasoline and ethanol.

Names	Chemical formula	Chemical structures	Molar mass (g/mol)	API gravities [a]	Heat combustion [b] (MJ/L)	$logP_{O/W}$ [c]	Fuel in water (%)
**Gasoline**	-	-	100-105	50-65	34.8	-	ND
**Ethanol**	C_2OH	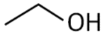	46.1	48	23.4	-	100
**n-Butanol**	C_4OH	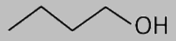	74.1	43	29.3	1.12	7.3
**n-Pentanol**	C_5OH	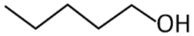	88.2	43	30.6	1.62	2.2
**i-Pentanol**	$3M\text{-}C_4OH$	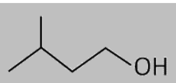	88.2	43	30.5	1.33	2.8
**n-Hexanol**	C_6OH	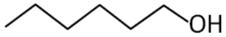	102.2	42	31.7	2.13	0.6
**Isoprenol**	$3M\text{-}3{=}C_4OH$	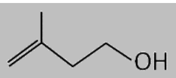	86.1	34	30.7	1.14	ND
**Isoprenol isomer 1**	$3M\text{-}2{=}C_4OH$	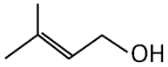	86.1	35	30.5	1.36	17
**Isoprenol isomer 2**	$2M\text{-}3{=}C_4OH$	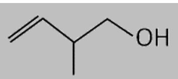	86.1	38	30.0	1.07	ND

Note: a) API gravities are calculated according to chemical density; b) heat combustion is calculated from the enthalpy of formation at 25 ℃[1]; and c) $logP_{O/W}$ is predicted at website www.molinspiration.com. ND indicates ‘not determined’.

Table S2. Mutant strains used in this study.

Names	Keio No.	MDT families	Names	Keio No.	MDT families
**BWΔ*macA***	JW0862	ABC	**BWΔ*cmr***	JW0826	MFS
**BWΔ*macB***	JW0863	ABC	**BWΔ*emrA***	JW2660	MFS
BWΔmdlA	JW0438	ABC	**BWΔ*emrB***	JW2661	MFS
**BWΔ*mdlB***	JW5061	ABC	**BWΔ*emrD***	JW5634	MFS
**BWΔ*mglB***	JW2137	ABC	**BWΔ*emrK***	JW2364	MFS
**BWΔ*rbbA***	JW5676	ABC	**BWΔ*emrY***	JW2365	MFS
**BWΔ*yddA***	JW5242	ABC	**BWΔ*fsr***	JW0468	MFS
**BWΔ*yojI***	JW2199	ABC	**BWΔ*hsrA***	JW3733	MFS
**BWΔ*acrA***	JW0451	RND	**BWΔ*mdtD***	JW2077	MFS
**BWΔ*acrB***	JJW0452	RND	**BWΔ*mdtG***	JW1040	MFS
**BWΔ*acrD***	JW2454	RND	**BWΔ*mdtH***	JW1052	MFS
**BWΔ*acrE***	JW3233	RND	**BWΔ*mdtL***	JW3688	MFS
**BWΔ*acrF***	JW3234	RND	**BWΔ*yajR***	JW5059	MFS
**BWΔ*cusA***	JW0564	RND	**BWΔ*ydeA***	JW1521	MFS
**BWΔ*mdtA***	JW5338	RND	**BWΔ*ydeE***	JW1527	MFS
**BWΔ*mdtB***	JW2060	RND	**BWΔ*ydiM***	JW1680	MFS
**BWΔ*mdtC***	JW2061	RND	**BWΔ*ydhC***	JW1652	MFS
**BWΔ*mdtE***	JW3481	RND	**BWΔ*yebQ***	JW5299	MFS
**BWΔ*mdtF***	JW3482	RND	**BWΔ*yjiO***	JW4300	MFS
**BWΔ*emrE***	JW0531	SMR	**BWΔ*ynfM***	JW1588	MFS
**BWΔ*mdtI***	JW1591	SMR	**BWΔ*mdtK***	JW1655	MATE
**BWΔ*mdtJ***	JW1592	SMR	**BWΔ*tolC***	JW5503	OMP
**BWΔ*sugE***	JW5738	SMR	**BWΔ*acrAB***	This study	-
**BWΔ*bcr***	JW5363	MFS	**BWΔ*ABC***	This study	-

Table S3. Cell growth of the MDT null mutants in the absence and presence of isoprenol.

Strains	Cell growth (OD_{600})		Strains	Cell growth (OD_{600})	
	No isoprenol	0.5% (v/v) isoprenol		No isoprenol	0.5% (v/v) isoprenol
BW25113	8.30 ± 0.16	4.12 ± 0.01	BWΔ*bcr*	7.94 ± 0.14	4.06 ± 0.01
BWΔ*macA*	8.08 ± 0.21	3.58 ± 0.01	BWΔ*cmr*	7.77 ± 0.05	4.03 ± 0.03
BWΔ*macB*	7.38 ± 0.09	3.00 ± 0.04	BWΔ*emrA*	7.28 ± 0.11	2.64 ± 0.06
BWΔ*mdlA*	7.93 ± 0.01	3.71 ± 0.04	BWΔ*emrB*	8.08 ± 0.17	4.50 ± 0.06
BWΔ*mdlB*	8.03 ± 0.13	3.57 ± 0.07	BWΔ*emrD*	8.45 ± 0.11	4.44 ± 0.04
BWΔ*mglB*	8.01 ± 0.17	3.89 ± 0.07	BWΔ*emrK*	7.59 ± 0.16	3.69 ± 0.18
BWΔ*rbbA*	7.98 ± 0.04	3.75 ± 0.22	BWΔ*emrY*	7.39 ± 0.06	3.43 ± 0.00
BWΔ*yddA*	8.03 ± 0.05	4.35 ± 0.01	BWΔ*fsr*	8.34 ± 0.01	4.36 ± 0.24
BWΔ*yojI*	7.68 ± 0.03	3.31 ± 0.04	BWΔ*hsrA*	8.21 ± 0.04	4.42 ± 0.01
BWΔ*acrA*	8.13 ± 0.03	6.26 ± 0.23	BWΔ*mdtD*	8.00 ± 0.04	3.98 ± 0.31
BWΔ*acrB*	8.28 ± 0.08	6.31 ± 0.13	BWΔ*mdtG*	7.42 ± 0.08	4.05 ± 0.01
BWΔ*acrD*	7.50 ± 0.14	3.13 ± 0.14	BWΔ*mdtH*	7.94 ± 0.15	4.23 ± 0.04
BWΔ*acrE*	7.57 ± 0.11	3.73 ± 0.04	BWΔ*mdtL*	7.78 ± 0.07	3.83 ± 0.12
BWΔ*acrF*	8.32 ± 0.11	4.45 ± 0.06	BWΔ*yajR*	7.64 ± 0.02	3.52 ± 0.04
BWΔ*cusA*	7.76 ± 0.01	3.75 ± 0.04	BWΔ*ydeA*	7.87 ± 0.06	4.22 ± 0.20
BWΔ*mdtA*	7.94 ± 0.14	4.27 ± 0.08	BWΔ*ydeE*	7.92 ± 0.10	3.82 ± 0.00
BWΔ*mdtB*	7.30 ± 0.30	2.95 ± 0.01	BWΔ*ydiM*	8.06 ± 0.05	3.22 ± 0.16
BWΔ*mdtC*	7.28 ± 0.06	2.93 ± 0.01	BWΔ*ydhC*	7.98 ± 0.03	3.80 ± 0.28
BWΔ*mdtE*	7.34 ± 0.05	3.68 ± 0.23	BWΔ*yebQ*	7.61 ± 0.02	3.50 ± 0.04
BWΔ*mdtF*	7.96 ± 0.13	4.06 ± 0.02	BWΔ*yjiO*	8.06 ± 0.05	4.05 ± 0.19
BWΔ*emrE*	7.47 ± 0.18	3.69 ± 0.36	BWΔ*ynfM*	8.55 ± 0.06	4.47 ± 0.30
BWΔ*mdtI*	7.41 ± 0.34	3.47 ± 0.33	BWΔ*mdtK*	7.68 ± 0.15	3.45 ± 0.00
BWΔ*mdtJ*	7.75 ± 0.06	2.99 ± 0.06	BWΔ*tolC*	8.16 ± 0.04	5.39 ± 0.08
BWΔ*sugE*	8.30 ± 0.14	4.25 ± 0.00			

Note: The results are presented as means ± standard divisions.

Table S4. Transcript profiles of targeted transporters in wild type *E. coli* BW25113 and mutants BWΔ*acrA*, BWΔ*acrA* and BWΔ*tolC* upon isoprenol exposure.

Genes	Transcript changes to isoprenol (fold)[a]	Transcript profiles in the mutants (fold)[b]		
		BWΔ*acrA*	BWΔ*acrB*	BWΔ*tolC*
acrA	3.16 ± 0.87		1.59 ± 0.08	1.32 ± 0.01
acrB	2.66 ± 0.36	1.97 ± 0.09		1.42 ± 0.13
tolC	1.48 ± 0.42	0.88 ± 0.09	0.81 ± 0.06	
emrA	1.52 ± 0.44	1.40 ± 0.14	1.11 ± 0.09	1.02 ± 0.08
macB	2.36 ± 0.41	1.02 ± 0.31	1.21 ± 0.33	1.02 ± 0.05
mdtC	1.63 ± 0.26	1.20 ± 0.45	1.25 ± 0.13	1.10 ± 0.01
mdtJ	1.98 ± 0.50	0.83 ± 0.11	1.02 ± 0.06	0.51 ± 0.05
acrD	1.65 ± 0.16	1.44 ± 0.15	1.91 ± 0.17	2.40 ± 0.16
ydiM	1.34 ± 0.41	1.24 ± 0.98	1.69 ± 1.42	0.67 ± 0.52

Note: a) *E. coli* BW25113 was grown in 2YT medium with 0.5% (v/v) of isoprenol at 30℃ for 6 h. Changes (folds) were normalized to those of the cultures without isoprenol; b) Three mutants were grown in 2YT medium with 0.5% (v/v) of isoprenol at 30℃ for 6 h. Transcript changes (folds) were normalized to *E. coli* BW25113. The results are presented as means ± standard divisions.

Table S5. Primers used for deletion of *acrAB*.

Names	Sequences (5' to 3')	References
**DacrAB -F**	ACTTTTGACCATTGACCAATTTGAAATCGGACACT CGAGGTTTACATATGATTCCGGGGATCCGTCGACC	NIG, Mishima, Japan
**DacrAB-R**	TTACGCGGCCTTAGTGATTACACGTTGTATCAATGA TGATCGACAGTATGTGTAGGCTGGAGCTGCTTCG	NIG, Mishima, Japan

Table S6. Primers used for quantitative PCR.

Names	Sequences (5' to 3')	Amplicon sizes	References
**QacrA-F**	CTTAGCCCTAACAGGATGTG	189 bp	2
**QacrA-R**	TTGAAATTACGCTTCAGGAT		
**QacrB-F**	CGTACACAGAAAGTGCTCAA	183 bp	2
**QacrB-R**	CGCTTCAACTTTGTTTTCTT		
**QtolC-F**	CCGGGATTTCTGACACCTCTT	89 bp	3
**QtolC-R**	TTTGTTCTGGCCCATATTGCT		
**QemrA-F**	CACCGGTAAAGTGGTTGGTC	156 bp	4
**QemrA-R**	ATACGCAGCGGATATTGCTC		
**QmacB-F**	GGCTGGAAGACCGTACAGAG	118 bp	4
**QmacB-R**	GTTGGTTCATCGGCAAGAAT		
**QmdtC-F**	ATCTCGATCCCGAAAACCTT	167 bp	4
**QmdtC-R**	CCTGTAAAGCCGGTGACATT		
**QmdtJ-F**	ATTAGGTCTGGCTATTGCTA	137 bp	5
**QmdtJ-R**	TAACGGCGAAAGAGAGAA		
**QacrD-F**	TCCTTGCTGGTGGTATTCCT	223 bp	4
**QacrD-R**	TGGCCTTTTTGGTTCATCTC		
**QydiM-F**	TTAGCCAGTTATACCTTATATGG	114 bp	This study
**QydiM-R**	GATCGACATAGTGTATGACATGC		
**QcysG-F**	TTGTCGGCGGTGGTGATGTC	105 bp	6
**QcysG-R**	ATGCGGTGAACTGTGGAATAAACG		

Table S7. Primers used for plasmid construction.

Names	Sequences (5' to 3')	References
**acrD-BamH-F**	GACGGATCCAAGAGGTCCTCTTTTAATGGCGAATTTC	This study
**acrD-Sal-R**	GATGTCGAC TTATTCCGGGCGCGGCTTCAGCGG	This study
**emrAB-BamH-F**	AGGATCCAGGAGAACAATATGAGCGCAAATGCG	This study
**emrAB-Sal-R**	GATGTCGAC TTAGTGCGCACCGCCTCCGCCG	This study
**macAB-Kpn-F**	GTGGTACCAGGGAGAAAATTTATGAAAAAGCGGAAAAC	This study
**macAB-Xba-R**	CTTCTAGAGAAGCGGCAGTCGCATAGC	This study
**mdtBC-Bgl-F**	TCGAGATCT AAGGAGCACGCTCCTGATGCAGGTGTTAC	This study
**mdtBC-Xho-R**	CTACTCGAG TTACTCGGTTACCGTTTGTTTAGGTTTACGC	This study
**mdtJI-BamH-F**	GACGGATCCTTGCAGGAGAAGGACAATGTATATTTATTG	This study
**mdtJI-Sal-R**	GATGTCGAC TTATCAGGCAAGTTTCACCATGATC	This study
**ydiM-BamH-F**	GACGGATCCAAGAGGTAGAACCTATGAAAAATCCCTATTTC	This study
**ydiM-Sal-R**	GATGTCGAC TGCATTACCCACCCGGAGCGAC	This study

Note: Restriction enzyme site are underlined.

Table S8. Plasmids used in this study.

Names	Descriptions	References
**pTrc99A**	P_{trc} promoter, pBR322 origin, *lacI*^{q}, and Amp^{r}	Amersham Bioscience
**pT-acrD**	pTrc99A containing *acrD* gene	This study
**pT-emrAB**	pTrc99A containing *emrAB* operon	This study
**pT-macAB**	pTrc99A containing *macAB* operon	This study
**pT-mdtBC**	pTrc99A containing *mdtBC* operon	This study
**pT-mdtJI**	pTrc99A containing *mdtJI* operon	This study
**pT-ydiM**	pTrc99A containing *ydiM* gene	This study
**pT-tolC**	pTrc99A containing *tolC* gene	7
**pKD13**	Template plasmid for gene disruption	8
**pCP 20**	Removal of kanamycin resistance cassette	8

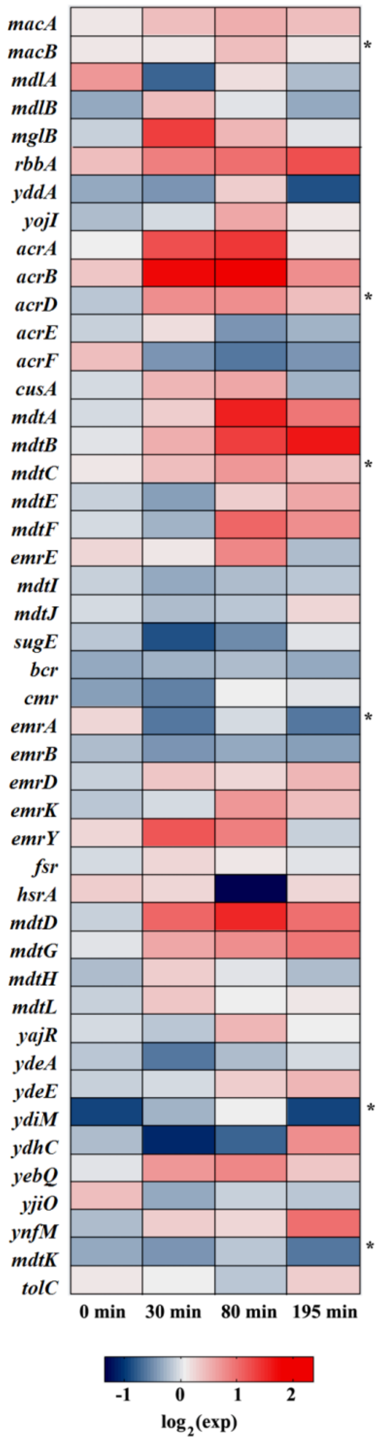

Figure S1. Microarray analysis of transporter responses to butanol. The data are adopted from Gene expression Omnibus (Accession No. GSE16973)[9]. Butanol was added at a concentration of 0.8% for a given time. Transcript change for each transporter gene was from three biological replicates. The asterisked (*) transporters indicate the identified transporters engaged in isoprenol extrusion in this study.

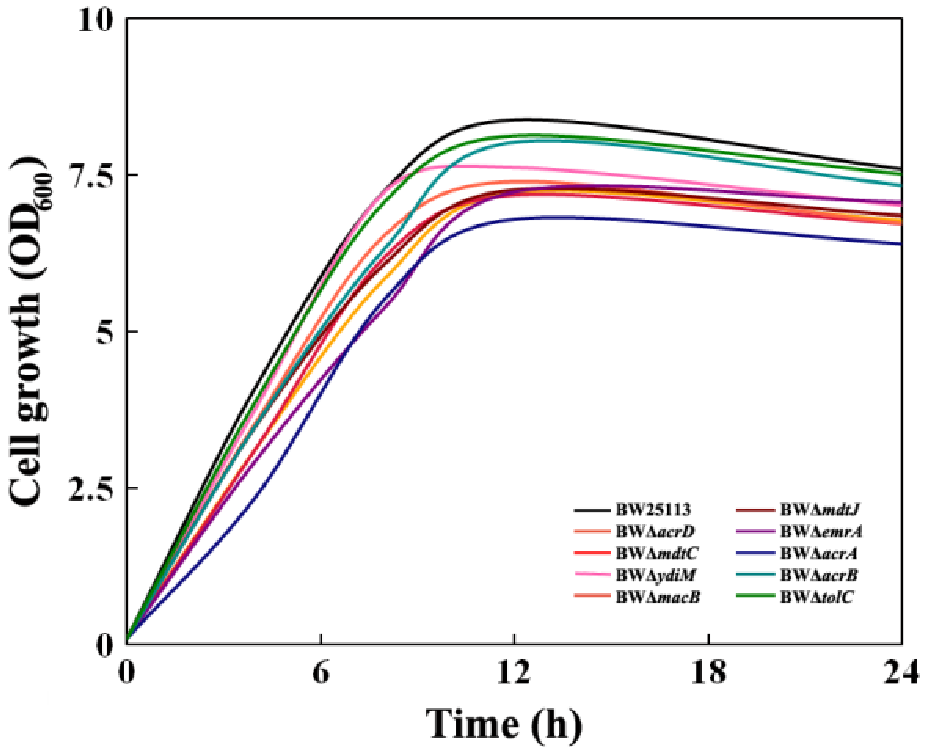

Figure S2. Cell growth of isoprenol susceptible and resistant mutants in the absence of isoprenol. Susceptible mutants BWΔ*acrD* (orange), BWΔ*emrA* (purple), BWΔ*macB* (tomato), BWΔ*mdtC* (red), BWΔ*mdtJ* (maroon) and BWΔ*ydiM* (pink); resistant mutants BWΔ*acrA* (blue), BWΔ*acrB* (cyan) and BWΔ*tolC* (green); and wild type *E. coli* BW25113 (black) were grown in 2YT medium at 30℃. Cell growth was measured every 6 h. Results are the means of two biological replicates.

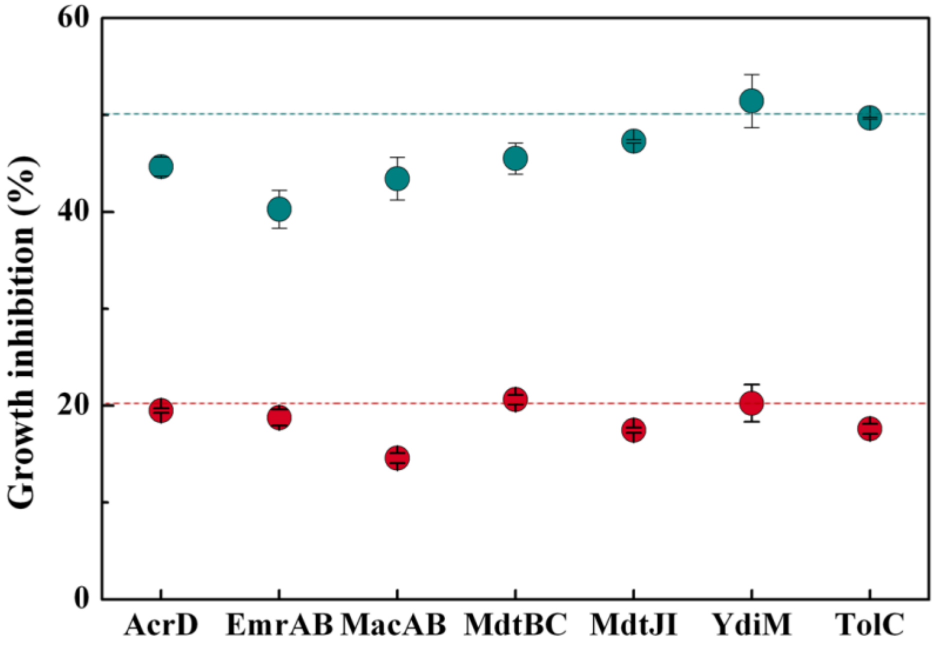

Figure S3. Growth inhibition by isoprenol on the strains expressing the identified transporters in the absence of IPTG. Growth inhibition was determined with *E. coli* BW25113 (dark cyan dots) and BWΔ*acrAB* (red dots) expressing AcrD, EmrAB, MacAB, MdtABC, MdtJI and YdiM. Growth inhibitions of *E. coli* BW25113 (dark cyan dashed-line) and BWΔ*acrAB* (red dashed-line) harboring an empty vector pTrc99A with no overexpression of these transporters was also measured as a control. All strains were grown in 2YT medium with 0.5% (v/v) isoprenol at 30℃ for 12 h. Error bars represent the standard deviation of two biological replicates.

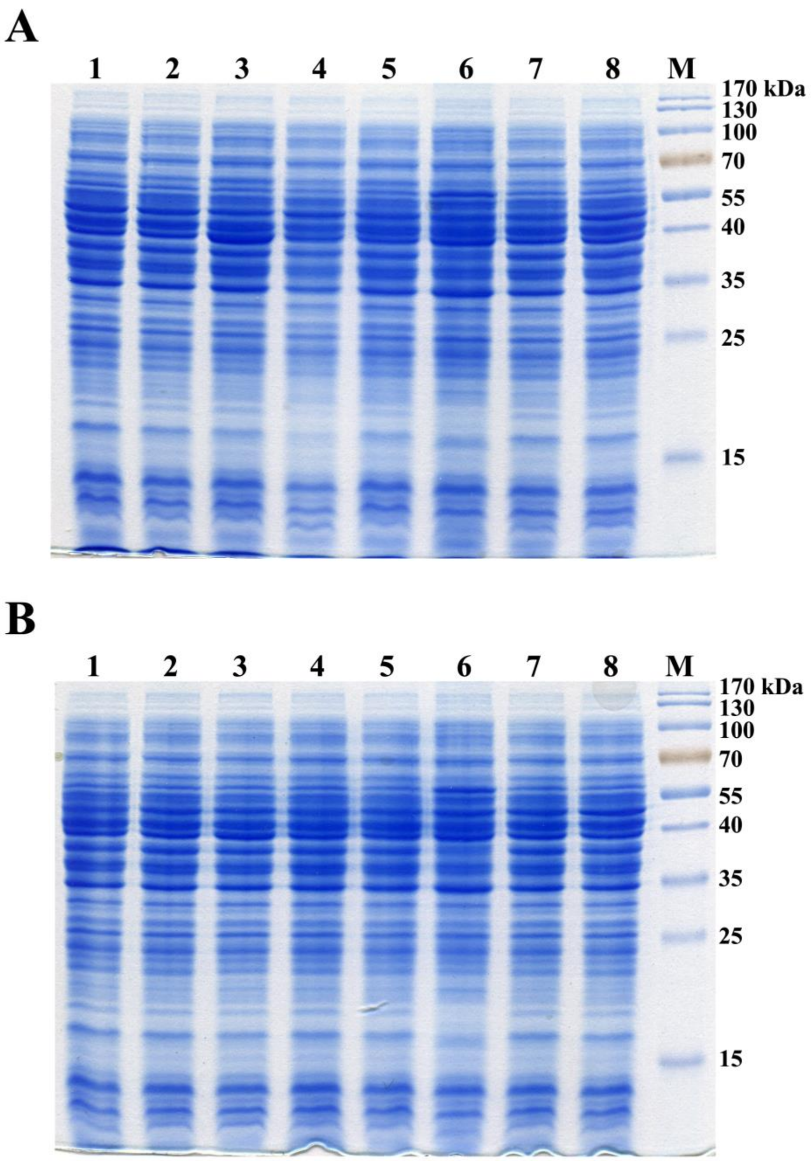

Figure S4. SDS-PAGE analysis of the identified transporters. (A) No IPTG induction, and **(B)** Induction with 0.2 mM of IPTG. *E. coli* BW25113 harboring pTrc99A (lane 1), pT-acrD (lane 2) pT-emrAB (lane 3), pT-ydiM (lane 4), pT-mdtJI (lane 5), pT-macAB (lane 6) pT-mdtBC (lane 7), and pT-tolC (lane 8) were grown in 2YT medium at 30℃ for 12 h. All strains were initially induced with 0.2 mM of IPTG. Theoretical molecular weight for each protein is 113 kDa (AcrD), 43 kDa (EmrA), 57 kDa (EmrB), 45 kDa (YdiM), 41 kDa (MacA), 70 kDa (MacB), 112 kDa (MdtB), 110 kDa (MdtC), and 54 kDa (TolC). Letter M indicates PageRuler Prestained Protein Ladder (Thermo Scientific, IL).

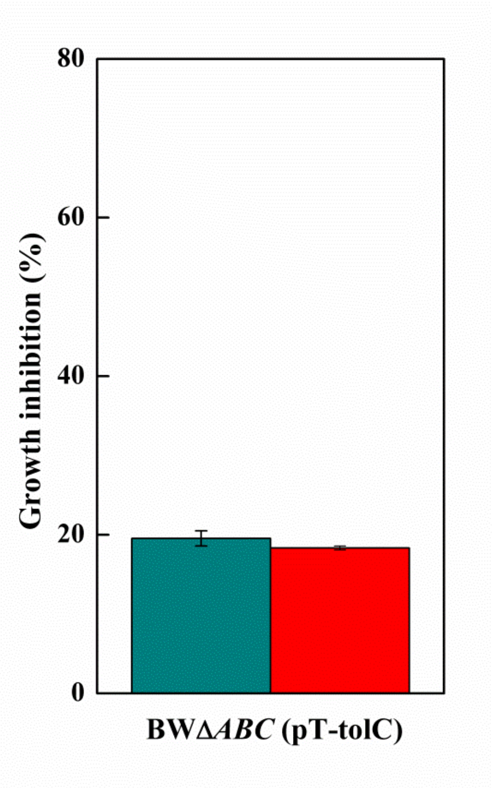

Figure S5. Growth inhibition by isoprenol on the strain BWΔ*ABC* expressing TolC. Growth inhibition was determined from BWΔ*ABC* expressing TolC in absence (dark cyan bar) and presence (red bar) of 0.2 mM of IPTG. The strain was grown in 2YT medium with 0.5% (v/v) isoprenol at 30℃ for 12 h. Error bars represent the standard deviation of two biological replicates.

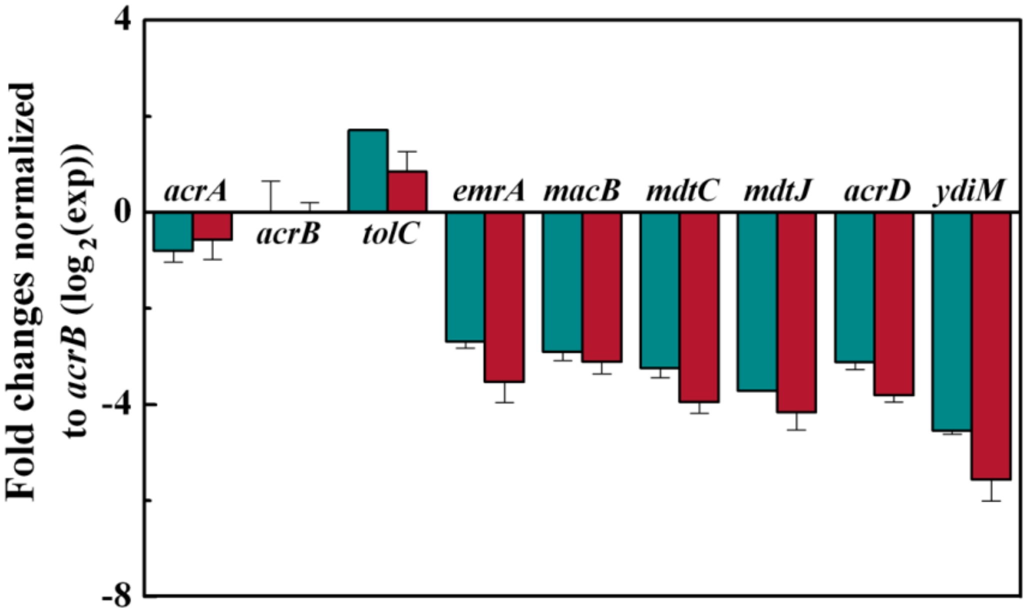

Figure S6. Relative abundances to transcripts of *acrB* transporter gene. *E. coli* BW25113 was grown in 2YT medium without (dark cyan bars) or with (red bars) 0.5% (v/v) of isoprenol at 30℃ for 6 h. Transcript changes (folds) were normalized to *acrB* gene. Results are the means of three biological replicates.

References

1 Lide, D. R. *CRC Handbook of Chemistry and Physics*. Internet Vession 2005 edn, (CRC Press, 2005).

2 Viveiros, M. *et al.* Antibiotic stress, genetic response and altered permeability of *E. coli*. *PLoS One* **2**, e365, doi:10.1371/journal.pone.0000365 (2007).

3 Lamikanra, A. *et al.* Rapid evolution of fluoroquinolone-resistant *Escherichia coli* in Nigeria is temporally associated with fluoroquinolone use. *BMC Infect. Dis.* **11**, 312, doi:10.1186/1471-2334-11-312 (2011).

4 Bohnert, J. A., Schuster, S., Fahnrich, E., Trittler, R. & Kern, W. V. Altered spectrum of multidrug resistance associated with a single point mutation in the *Escherichia coli* RND-type MDR efflux pump YhiV (MdtF). *J. Antimicrob. Chemother.* **59**, 1216-1222, doi:10.1093/jac/dkl426 (2007).

5 Zhou, K., Zou, R., Stephanopoulos, G. & Too, H. P. Metabolite profiling identified methylerythritol cyclodiphosphate efflux as a limiting step in microbial isoprenoid production. *PLoS One* **7**, e47513, doi:10.1371/journal.pone.0047513 (2012).

6 Zhou, K. *et al.* Novel reference genes for quantifying transcriptional responses of *Escherichia coli* to protein overexpression by quantitative PCR. *BMC Mol. Biol.* **12**, 18, doi:10.1186/1471-2199-12-18 (2011).

7 Shah, A. A. *et al.* Enhancement of geraniol resistance of *Escherichia coli* by MarA overexpression. *J. Biosci. Bioeng.* **115**, 253-258, doi:10.1016/j.jbiosc.2012.10.009 (2013).

8 Datsenko, K. A. & Wanner, B. L. One-step inactivation of chromosomal genes in *Escherichia coli* K-12 using PCR products. *Proc. Natl. Acad. Sci. U S A* **97**, 6640-6645, doi:10.1073/pnas.120163297 (2000).

9 Rutherford, B. J. *et al.* Functional genomic study of exogenous *n*-butanol stress in *Escherichia coli*. *Appl. Environ. Microbiol.* **76**, 1935-1945, doi:10.1128/AEM.02323-09 (2010).
